# Expression Analysis of Autophagy Related Markers LC3B, p62 and HMGB1 Indicate an Autophagy-Independent Negative Prognostic Impact of High p62 Expression in Pulmonary Squamous Cell Carcinomas

**DOI:** 10.3390/cancers10090281

**Published:** 2018-08-21

**Authors:** Rupert Langer, Christina Neppl, Manuel D. Keller, Ralph A. Schmid, Mario P. Tschan, Sabina Berezowska

**Affiliations:** 1 Institute of Pathology, University of Bern, Murtenstrasse 31, 3008 Bern, Switzerland; rupert.langer@pathology.unibe.ch (R.L.); christina.neppl@pathology.unibe.ch (C.N.); manuel.keller@students.unibe.ch (M.D.K.); mario.tschan@pathology.unibe.ch (M.P.T.); 2 Division of General Thoracic Surgery, Inselspital University Hospital Bern, 3010 Bern, Switzerland; ralph.schmid@insel.ch; 3 Department of Clinical Research, University of Bern, Murtenstrasse 35, 3008 Bern, Switzerland

**Keywords:** Lung cancer, squamous cell carcinoma, autophagy, LC3B, p62, HMGB1

## Abstract

Autophagy is involved in maintaining cellular homeostasis under stress conditions. It also plays an important role in various diseases including cancer. Pulmonary squamous cell carcinomas (pSQCC) at present lack targetable molecular alterations, and demand alternative therapeutic options. We assessed the expression levels of autophagy related proteins LC3B, p62, and HMGB1 in 271 primary resected pSQCC by immunohistochemistry, in correlation with clinical and pathological parameters, as a rationale for a potential autophagy directed therapy. LC3B, p62, and HMGB1 staining showed various patterns. LC3B^high^p62^low^ levels, suggested to indicate intact activated autophagy, were associated with prolonged disease specific survival (DSS) and LC3B^high^p62^high^ levels, indicating activated but late stage impaired autophagy, with shorter DSS (*p* = 0.024). p62^high^ expression regardless of LC3B, however, showed an even stronger association with shorter DSS (*p* = 0.015) and was also an independent negative prognostic factor in multivariate analysis (HR = 2.99; 95% CI 1.38–6.52; *p* = 0.006). HMGB1 expression correlated neither with the expression of LC3B and p62, nor with patients’ outcome. Different states of autophagy characterized by distinct p62 and LC3B expression patterns may be linked to patient’s prognosis in pSQCC. Our results, however, point also to an autophagy independent role of p62 with an even more pronounced prognostic impact compared to autophagy related p62.

## 1. Introduction

Non-small cell lung cancer (NSCLC), the largest subgroup of pulmonary carcinomas, belongs in the category of the most frequent malignant diseases worldwide [1]. Accurate histopathologic and molecular tumor characterization is the base of individualized treatment in preoperative, postoperative, and metastatic stages of the disease. Adenocarcinomas harbor alterations allowing a more detailed tumor classification according to their mutational status, which also represents targets for treatment. In contrast, antitumoral therapy targeting specific molecular alterations is unavailable in pulmonary squamous cell carcinomas (pSQCC) [2], although some genotypic characteristics present potential actionable targets for treatment [3]. Moreover, only a subset of pSQCC responds to the highly effective anticancer treatment by immune checkpoint inhibition [4,5].

Autophagy, with its best-known subtype of macro-autophagy, is a catabolic cellular mechanism responsible for the degradation of cellular components, which contributes to the maintenance of cellular integrity under stress conditions [6,7]. Dysfunctional autophagy is observed in the context of neurodegenerative or autoimmune diseases [8,9,10], as well as cancer [11,12]. Autophagosomes are structures with double layer membranes that engulf organelles and cellular components for subsequent degradation and represent the functional and structural hallmark of autophagy. Proteins that play a role in the formation of autophagosomes have been discussed to harbor the potential to visualize autophagy in tissue samples [13,14]. The microtubule-associated protein 1 light chain 3 B (LC3B), in its cytosolic LC3B-I form is transformed to the lipidated LC3B-II while forming an integral part of the membranes of the autophagosomes. p62/sequestosome 1 (SQSTM1) interacts with LC3B, targeting ubiquitinated substrates to autophagosomes [14,15]. Recently, the stress-associated protein, high-mobility group protein-B1 (HMGB1), that partially serves as a regulator of autophagy has been described as harboring a prognostic impact in cancer, in particular in association with LC3B [16]. Given its high relevance for cancer development and progression, but also for therapy resistance, autophagy has been widely considered an interesting potential target for specific, molecular based anti-cancer treatment [17,18,19,20].

Following a preliminary study on early-stage NSCLC comprising both adenocarcinomas and pSQCC [21] we investigated the relevance of LC3B, p62/SQSTM1 (short p62), and HMGB1 expression in a well-characterized cohort of pSQCCs [22] as a potential rationale for autophagy directed therapy in pSQCC.

## 2. Results

### 2.1. LC3B, p62 and HMGB1 Expression in Pulmonary Squamous Cell Carcinoma

**Figure 1:**
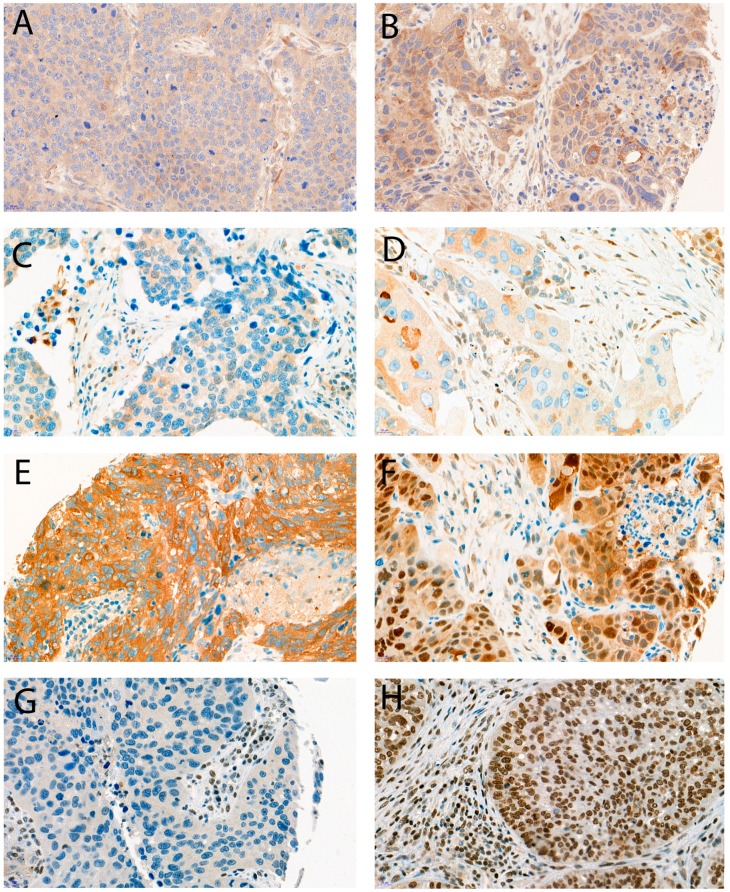
Examples of immunohistochemical stainings; (**A**) LC3B low, (**B**) LC3B high, (**C**) p62 low (any cellular compartment), (**D**) p62 cytoplasmic low, (**E**) p62 cytoplasmic/dot like high, nuclear low, (**F**) p62 cytoplasmic/dot like low, nuclear high, (**G**) HMGB1 low, (**H**) HMGB1 high. Objective magnification, 40×.

Dot-like LC3B staining and dot-like, cytoplasmic, and nuclear p62 staining were observed in various levels in the tumor cells of the 271 pSQCCs, as shown in Figure 1. For the single scores see supplemental material Tables S1–S5. After setting the optimal cut-offs as described below, low LC3B dot-like staining was observed in 123 cases (45.4%), and 148 cases (54.6%) had high levels. Combined dot-like/cytoplasmic staining levels were low in 111 cases (41.0%) and high in 160 cases (59.0%). Nuclear staining for p62 was low in 157 cases (57.9%) and high in 114 cases (42.1%). Similarly, HMGB1 nuclear staining was observed at all levels. We observed low HMGB1 expression in 153 cases (56.5%) and high expression in 118 cases (43.5%).

There was a significant correlation between LC3B dot-like staining and p62 dot-like/cytoplasmic staining (*p* = 0.036) and an inverse correlation between p62 dot-like/cytoplasmic staining and p62 nuclear staining (*p* < 0.001). No significant correlations were found between LC3B, p62, and HMGB1 staining patterns.

### 2.2. Correlation of LC3B, p62, and HMGB1 Expression with Pathologic Features

**Figure 2:**
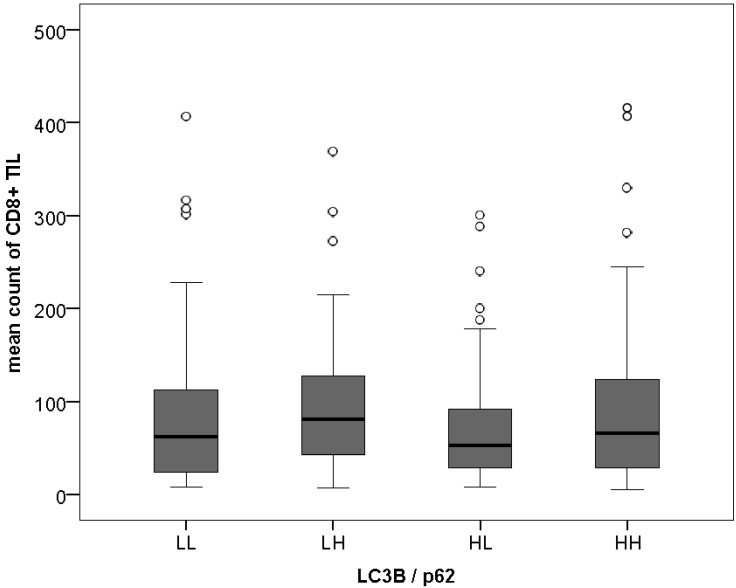
LC3B/p62 patterns and CD8+Tumor-infiltrating Lymphocytes. LL = LC3B^low^p62^low^; LH = LC3B^low^p62^high^; HL = LC3B^high^p62^low^; HH = LC3B^high^p62^high^, ° indicate outliers.

Higher LC3B levels were observed more frequently in tumors with better differentiation (*p* = 0.028), as well as high-grade p62 nuclear staining (*p* < 0.001). Associations between LC3B, p62, and HMGB1 staining patterns and pathological features such as the extend of the primary tumor (pT category), the presence of lymph node metastases (pN category), or the tumor stage as defined by the tumor-node-metastasis (TNM) classification of the Union for International Cancer Control and American Joint Committee on Cancer (UICC/AJCC TNM stage) [23] were not found (see supplemental Tables S1–S5 for detailed results). Of note, there was no association between single and combined LC3B, p62, and HMGB1 staining patterns and tumor infiltrating lymphocyte (TIL) counts for CD8+ T-cells, as shown in Figure 2, nor CD3+ T-cells.

### 2.3. Combination of LC3B and p62

Dot-like/combined dot-like and cytoplasmic LC3B^high^p62^low^ staining, suggested to be indicative of intact activated autophagy [24], was observed in 52 cases (19.2%), and dot-like/dot-like and cytoplasmic LC3B^high^p62^high^ expression, indicative of activated but late-stage impaired autophagy, was observed in 96 cases (35.4%). Basal (i.e., low) levels of LC3B and p62 were seen in 59 cases (21.8%) and LC3B^low^p62^high^ pattern in 64 cases (23.6%). Poorly differentiated tumors significantly more frequently showed a LC3B^low^p62^high^ pattern in comparison to the other combinations (*p* = 0.023). As for the single markers, no further significant association between this “autophagy” classifier and other pathological features, including TIL counts was found.

### 2.4. Survival Analysis

**Figure 3:**
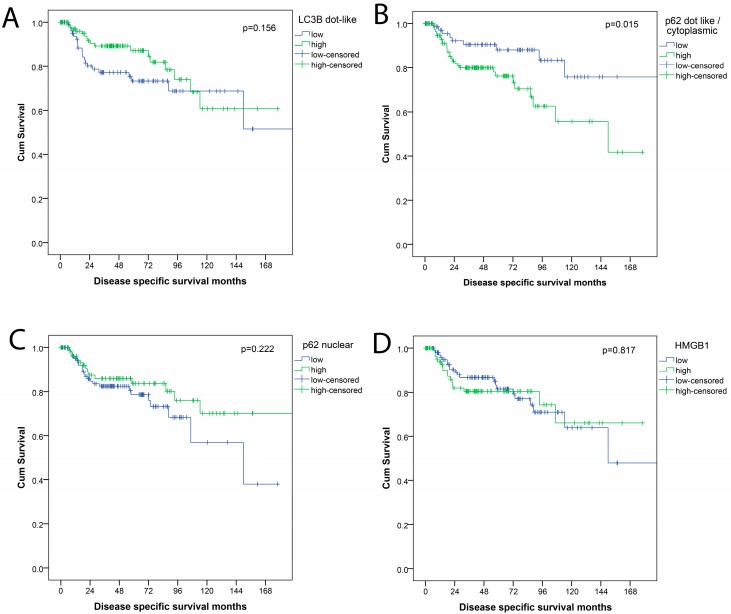
Survival curves (disease free survival) for expression of autophagy related proteins (**A**) LC3B; (**B**) p62 dot-like/cytoplasmic; (**C**) p62 nuclear; (**D**) HMGB1.

Detailed survival data were available for 203 patients. Mean disease specific survival (DSS) was 140 months (95% CI: 124–156 months). Mean time to relapse (TTR) was 106 months (95% CI: 92–121 months). Mean overall survival (OS) was 86 months (95% CI: 74–98 months), but not used for further analysis for the reasons explained below. Pathologic parameters that were associated with prognosis (DSS; TTR) were pT-category (*p* < 0.001; *p* = 0.001), pN-category (*p* = 0.016), presence of distant metastases (*p* < 0.001; *p* = 0.078), UICC/AJCC TNM stage (*p* = 0.001; *p* = 0.014) and resection status (*p* < 0.001 each). Age, gender, and tumor differentiation (grading) were not associated with outcome. In single analysis of the autophagy markers, high LC3B levels were only in trend associated with a better outcome for patients (DSS *p* = 0.156; TTR *p* = 0.329). For p62, high dot-like/cytoplasmic staining patterns were associated with a worse outcome for patients (DSS *p* = 0.015; TTR *p* = 0.158). High levels of p62 nuclear staining were not significantly related to patients’ outcome, neither were HMGB1 levels, as shown in Figure 3.

**Figure 4:**
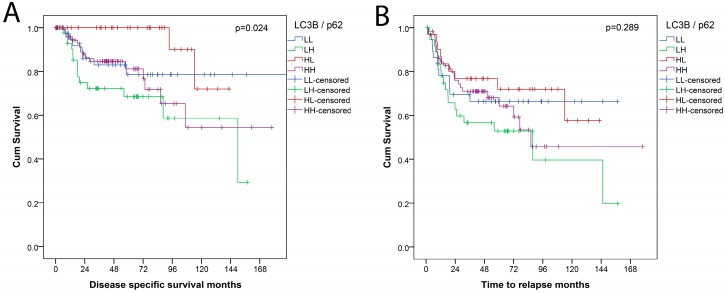
Survival curves for combination LC3B/p62 categorization into four subgroups: LL = LC3B^low^p62^low^; LH = LC3B^low^p62^high^; HL = LC3B^high^p62^low^; HH = LC3B^high^p62^high^; (**A**) disease specific survival (DSS); (**B**) time to relapse (TTR).

Stratification according to the “autophagy” combination LC3B/p62 showed an overall significant prognostic relevance (*p* = 0.024) with LC3B^high^p62^low^ pattern, indicative of activated, intact autophagy being associated with favorable DSS. In contrast, dot-like/dot-like and cytoplasmic LC3B^high^p62^high^ expression, indicative of activated but late-stage impaired autophagy was associated with worse DSS in this grouping. For TTR, the differences between the subgroups were not significant, as shown in Figure 4. In a separate analysis, the positive prognostic value of a LC3B^high^p62^low^ pattern was significant when compared to all other combinations (*p* = 0.021).

Multivariate analysis for DSS encompassing the prognostically most relevant factors pT category, pN category, presence of distant metastases, resection status and p62 dot-like/cytoplasmic staining demonstrated an independent prognostic value of p62 dot-like/cytoplasmic staining (hazard ratio, HR = 2.85; 95% CI: 1.30–6.25; *p* = 0.009; Table 1). This was also demonstrated in a model including UICC/AJCC TNM stage instead of the single pathological categories, see Table 2. Incorporating a LC3B^high^p62^low^ pattern instead of p62 alone did not show statistical significance (HR = 0.252; 95% CI: 0.60–1.06; *p* = 0.060 and HR = 0.249; 95% CI = 0.059–1.047; *p* = 0.058; respectively). Separate analysis of the patient subset treated with adjuvant chemo- or radiotherapy did not reveal significant differences.

## 3. Discussion

In this tissue-based study, we examined the impact of the expression of autophagy-related proteins LC3B, p62, and HMGB1 in a consecutive cohort of squamous cell carcinomas of the lung. Autophagy is a fascinating, complex catabolic process that enables cells to survive under stress conditions by degradation and self “digestion” of organelles and other cellular components. In healthy cells, autophagy maintains normal cellular metabolism, prevents inflammation, oxidative stress and DNA damage by removing damaged organelles and proteins. The role of autophagy in cancer has been discussed to be divergent [25]: In early stages, autophagy may show tumor-suppressing properties due to the supportive effects on non-neoplastic tissue. In late stages, however, autophagy may allow survival, dormancy, growth, and metastasis as it may provide alternative energy sources for tumor cells, thereby acting as a tumor promoter [9,26]. For cancer research and treatment, autophagy has gained major attention, since it is also considered a target for molecular-based tumor therapy [27]. Autophagy blockers, such as chloroquine, that are already widely used in different contexts, have been tested for their propensity for cancer treatment. In fact, chloroquine and hydroxychloroquine are most widely used in clinical trials, in the adjuvant and neoadjuvant setting and most often as additives to standard chemotherapy, radiotherapy, or targeted therapeutic approaches. Other substances that influence autophagy are also emerging in the field. We refer to excellent current reviews for a more comprehensive coverage of ongoing clinical trials [20,28]. For pSQCC that do not harbor targetable mutations, modulating autophagy may represent a potential therapeutic option.

We showed that (a) the expression of LC3B, p62, and HMGB1, which are important proteins involved in autophagy, can be visualized by immunohistochemistry, that (b) different expression levels in different subcellular compartments can be observed, and that (c) a LC3B^high^p62^low^ staining pattern is associated with better prognosis in univariate models, but does not persist in a multivariate model. This is in contrast to findings in other tumor types such as oral squamous cell carcinomas, where similar expression patterns were associated with a worse outcome [24]. In gastrointestinal cancers, they were not linked to prognosis [29,30]. Monitoring autophagy in tissue, however, is limited by the natural character of this process, which is a flux rather than a steady state [6,31]. In contrast to in vitro studies, guidelines for studying autophagy in human and mammalian tissue are limited [14,32,33]. Immunohistochemistry is, however, the methodology of choice, as it allows the assessment of the expression of autophagy-related proteins within the relevant histological context. It is important to validate the markers for sensitivity and specificity, as we have done in preparation for the current study [34]. For LC3B, in particular, only dot-like staining should be considered as autophagy related in view of its subcellular location and function during autophagosome formation. LC3B positive dots are established as surrogate markers for autophagosomes [33,34]. High dot-like LC3B levels, however, can also be expected when the autophagy flux is blocked or impaired at late stages of the process. A combination of dot-like LC3B levels with dot-like and cytoplasmic staining patterns of p62 expression, which should be elevated in early stages and lowered in late stages of intact autophagy, has been suggested to deliver an approximate snap-shot of autophagy status in tissue analysis with a LC3B^high^p62^low^ pattern indicating activated, intact autophagy [24,30]. In our study, this pattern was associated with a favorable outcome in univariate analysis. In this context, activated autophagy is not necessarily associated with tumor aggressiveness, but may rather reflect a high-stress status of the tumor, which may be responsible for better prognostic conditions.

HMGB1 is one of the most abundant chromatin-binding proteins and has been shown to play oncogenic roles in tumorigenesis of different tumor types including lung cancer [35]. Moreover, it interacts with and regulates autophagy in early stages. A combination of high HMGB1 and LC3B showed a correlation with a good prognosis in breast cancer, particularly after adjuvant chemotherapy [16]. In our study, however, the expression of HMGB1 was not associated with any clinical or pathological parameter, and the combination with LC3B did not show any prognostic relevance.

The most significant prognostic impact, however, was observed for a combined dot-like and cytoplasmic expression of p62, independent from LC3B status, with p62^high^ levels showing a significant association with worse outcome. p62 was also an independent prognostic factor in multivariate analysis. This is in line with the observations of a prior study on a mixed case collection of early-stage adeno- and squamous cell carcinomas of the lung [21] and can be considered as a true validation of the results of this previous study. Similar results with a negative prognostic impact of high p62 levels were also obtained by other groups from tissue analysis of NSCLCs [36] and lung adenocarcinomas [37]. Moreover, high p62 levels have been shown to be associated with more aggressive tumor behavior in several other tumor types [38]. It should be noted, however, that p62 is also involved in other pathways such as the Kelch-like ECH-associated protein 1 (KEAP1)/NF-E2-related factor 2 (NRF2) stress response pathway [39,40,41], nuclear factor kappa-B (NF-kappaB) or endoplasmatic reticulum stress response [42], and these pathways may be linked to or independent from autophagy. Therefore, it may not be appropriate to design p62 as an autophagy-specific biomarker.

High-level nuclear staining for p62 was observed in nearly half of the cases, associated with a better tumor differentiation, but showed no correlation with other pathological or clinical parameters including survival. The nuclear function of p62 is not well-characterized, and may also be primarily non-related to autophagy, although the mechanisms allowing shuttling between cytosol and nucleus have been described [43]. In a recent study on esophageal adenocarcinomas, we observed that nuclear p62 showed an influence on response to chemotherapy in vitro, but to a lesser degree compared to cytoplasmic p62. Ex vivo, low LC3B dot-like staining, high p62 cytoplasmic/dot-like staining and low p62 nuclear staining were associated with worse overall survival after neo-adjuvant treatment and surgery in these tumors [44]. Thus, nuclear p62 may play a particular role in cancer but it is very likely that the effects of nuclear p62 are both context and tumor type dependent as seen in other studies on different cancer entities [21,29,30]. Nevertheless, p62 with its complex regulatory network, cellular domains, and different intracellular localizations in addition to its multiple involvements in cellular processes including autophagy and apoptosis represents a highly promising target for innovative anti-cancer therapy [45].

Moreover, autophagy itself is regulated and influenced by a large number of different proteins and embedded in a variety of molecular networks, pathways, and biological processes [7,10]. The immunology of the tumor-host interaction, for example, has been shown to interact with autophagy [46] as seen in tumors such as breast cancer [47] or melanoma [48,49]. Interestingly, none of the autophagy related proteins included in the present study showed an association with the density of intratumoral T lymphocytes. Although this does not support the interaction between tumor immunity and autophagy, the crosstalk between these two players may reveal further perspectives for potent antitumor treatment.

## 4. Materials and Methods

### 4.1. Patient Cohort

A consecutive cohort of patients with primary resected pSQCC, diagnosed at the Institute of Pathology, University of Bern, between January 2000–December 2013 was investigated. The study was performed according to the REMARK-guidelines and was approved by the Cantonal Ethics Commission of the Canton of Bern (KEK 200/14), which waived the requirement for written informed consent. Initially, 402 patients met the inclusion criteria of the diagnosis pSQCC according to the pathology records [22]. Next, immunohistochemical stainings for p40 and TTF-1 definitively confirmed the squamous differentiation of the tumors later included in the study. Furthermore, the slides of all tumors were reevaluated regarding stage-relevant characteristics (such as pleural invasion) and grading. Grading was performed as described elsewhere [50]. In short: Grade 1 tumors showed prominent keratinization, easily visible intercellular bridges and prominent cell membranes. Grade 2 tumors had only scattered foci of keratinization, less frequent intercellular bridges, smaller sized tumor cells and could show comedo-like necrosis. Grade 3 tumors were poorly differentiated, with rare or missing intercellular bridges and lack of keratinization. Grade 1 and 2 tumors corresponded to keratinizing pSQCC, Grade 3 to non-keratinizing pSQCC according to the World Health Organization (WHO) classification of pSQCC [51]. As only three tumors showed grade 1 features, as shown in Table 3, Grade 1 and Grade 2 tumors were fused into the group of keratinizing pSQCC for further evaluation. Cases with a previous or concomitant diagnosis of primary SQCC of other organ systems, which would have raised the possibility of a metastatic lung disease rather than a primary carcinoma, and patients who had been treated with neoadjuvant therapy according to a reevaluation of clinical files were excluded. Cases already analyzed for LC3B and p62 in our previous study were also excluded [21]. The tumors were restaged according to the 8th edition of the UICC TNM-classification [23]. The case collection finally comprised 271 tumors from stages UICC I–III. Detailed clinico-pathological characteristics are provided in Table 3. Adjuvant chemotherapy or radiotherapy was administered in 102 patients (37.6%).

### 4.2. Next-Generation Tissue Microarray

Immunohistochemical stainings were applied on a next-generation tissue microarray (ngTMA) that was constructed with digital annotation of scanned slides and automatic transferal of the punches, as previously described [22,52]. Two separate ngTMA with a total of four punches per tumor (diameter = 0.6 mm) randomly taken from different tumor regions were used.

### 4.3. Immunohistochemical Staining and Scoring

Immunohistochemical stainings for LC3B and p62 were performed on 4 µm sections using an automated immunostainer Leica Bond RX (Leica Biosystems, Heerbrugg, Switzerland) with the following conditions (dilution, antigen retrieval), as described before [34]: LC3B (Novus Biologicals, Zug, Switzerland, rabbit polyclonal, #NB600-1384): 1:4000, tris buffer, 95 °C, 30 min; p62 (LabForce mbl, Nunningen, Switzerland, rabbit polyclonal, #PM0045): 1:8000, citrate buffer, 100 °C, 30 min; HMGB1 (Thermo scientific, Waltham, MA, USA, rabbit polyclonal, #PA116926): 1:800, citrate buffer, 100 °C, 30 min. For visualization, the Bond Polymer Refine Detection kit (Leica Biosystems, Muttenz, Switzerland, DS9800) was used according to the instructions of the manufacturer. Scoring of immunohistochemical staining patterns for LC3B and p62 detected in various subcellular components of the tumor cells was performed across all respective TMA cores as described before [21]: dot-like staining was scored from 0 to 3 as follows: Score 0—no dots or barely dots visible in <5% of the tumor cells, score 1—dots in 5–25% of the tumor cells, score 2—dots in 25–75% of the tumor cells, score 3—dots in >75% of the tumor cells. p62 cytoplasmic staining was scored from 0 to 3 as follows: Score 0—no or faint staining, score 1—weak staining, score 2—moderate staining visible and score 3—strong staining. p62 nuclear immunohistochemical staining was scored from 0 to 1 as follows: Score 0—nuclear staining visible in <10% of nuclei and score 1—nuclear staining visible in >10% of nuclei. Nuclear HMGB1 expression was scored as described in [16] with slight modifications: Negative (score 0), weak positive ≤50% (score 1), strong positive >50% (score 3), strong positive >80% (score 3). Examples of dot-like LC3B, dot-like, cytoplasmic and nuclear p62 and nuclear HMGB1 immunohistochemical stainings are shown in Figure 1. Scoring was performed by one experienced pathologist (RL) on a Zeiss Axioscope microsope at 40× objective magnification. For the purpose of correlation with clinico-pathological features, the immunhistochemical scores were then further categorized as either low or high as described before [21,29], and according to the prognostic value of the single values (the detailed survival curves are provided in supplemental Figure S1). LC3B dot-like staining was classified as low for score 0, and high for scores ≥1. Dot-like and cytoplasmic p62 staining was categorized as low for scores 0–1 and high for scores 2–3. Combined dot-like and cytoplasmic staining for p62 was classified as low for a sum score of 0–2 and high for a sum score of 3 and greater of the raw values, showing the best prognostic discrimination. A p62 nuclear staining score of 0 was classified as low and a score of 1 as high. HMGB1 expression was classified as low for scores 0 and 1, and high for scores 2 and 3, in accordance to published data [47].

### 4.4. Subclassification According to Autophagy Status

For the characterization of autophagy, several combination patterns of autophagy related proteins have been described in literature [33]. Following preliminary work of our group [29,30] and others [24] a combination of dot-like LC3B and p62 dot-like/cytoplasmic staining stratified the cases into four subtypes of autophagy states: Low LC3B dot-like/low p62 dot-like-cytoplasmic staining (LL), indicating basal autophagy; low LC3B dot-like/high p62 dot-like-cytoplasmic staining (LH), indicating low basal autophagy (early-stage impaired autophagy with a low number of autophagosomes, but accumulation of p62) or autophagy independent; high LC3B dot-like/low p62 dot-like-cytoplasmic staining (HL), indicating activated, intact autophagy and high LC3B dot-like/high p62 dot-like-cytoplasmic staining (HH), indicating activated autophagy, impaired at late stages [24].

### 4.5. Tumor Infiltrating Lymphocytes

For the analysis of a potential association between the autophagy markers and the inflammatory host response, we also included data for tumor infiltrating lymphocytes (TILs, CD3+ and CD8+ T lymphocytes) from a previous study that had been assessed on the same ngTMAs using digital image analysis [22].

### 4.6. Statistical Analysis

For statistical analysis, IBM SPPS Statistics 24 (IBM Corporation, Armonk, USA) was used. Group comparisons were performed using crosstabs, χ^2^-tests, and Fisher’s exact tests. Survival analysis encompassed time to recurrence (TTR), which was measured from the day of resection to loco-regional or metastatic recurrence or disease specific death. Disease specific survival (DSS) was determined from the time of resection to disease specific death. Due to a heavy bias regarding specifically tumor related survival issues (a significant number of deaths unrelated to pulmonary SQCC in our cohort, as discussed before more in detail [22]), overall survival (OS) and disease-free survival (DFS) were not calculated for the purpose of this study. Kaplan-Meier curves and log-rank tests were used for univariate survival analysis. For multivariate analysis, Cox regression analysis was used. The significance level for all statistical tests was set for a *p*-value of <0.05.

## 5. Conclusions

In conclusion, our tissue-based ex vivo data suggest that activated intact or activated late-stage impaired autophagy characterized by dot-like/dot-like cytoplasmic LC3B^high^p62^low^ or LC3B^high^p62^high^ expression levels occurs in a substantial subset of primary resected pSQCC and is associated with patients’ prognosis. Our results, however, also point towards a most likely autophagy-independent role of p62 in pSQCC with an association between high expression levels and unfavorable prognosis. This still encourages a potential clinical benefit of autophagy modulation in pSQCC, as autophagy plays a major role in degradation of p62, thereby constraining p62-dependent pathways that increase tumor aggressiveness. Following this thread of thought, patients could potentially benefit from increased autophagy in tumor cells. Our data undermine the need for further mechanistic studies in that regard.

## Figures and Tables

**Table 1:** Results of multivariate analysis including pT, pN, and M category.

Factor	HR	95.0% CI for HR		*p*-Value
		Lower	Upper	
pT category	1.282	0.981	1.675	0.069
pN category	1.364	0.828	2.247	0.223
pM category	2.511	0.307	20.532	0.390
R-status	2.555	1.107	5.899	0.028
p62 dots/cytoplasmic	2.854	1.303	6.251	0.009

**Table 2:** Results of multivariate analysis including UICC/AJCC TNM stage.

Factor	HR	95.0% CI for HR		*p*-Value
		Lower	Upper	
UICC/AJCC TNM stage	1.144	0.898	1.459	0.276
R-status	3.683	1.626	8.347	0.002
p62 dots/cytoplasmic	2.999	1.379	6.520	0.006

pT, extend of the primary tumor; pN, regional lymph node metastases; M, distant metastases; UICC/AJCC TNM stage, tumor stage as defined by the tumor-node-metastasis (TNM) classification of the Union for International Cancer Control and American Joint Committee on Cancer [23]; HR, hazard ratio.

**Table 3:** Description of the case collection.

Parameter		*n*	%
**Gender**	Male	225	83.0
	Female	46	17.0
**Age**	Median 69 years (43–85 years)		
**pT UICC 2017**	pT1a	4	1.5
	pT1b	19	7.0
	pT1c	33	12.2
	pT2a	54	19.9
	pT2b	39	14.4
	pT3	64	23.6
	pT4	58	21.4
**pN UICC 2017**	pN0	137	50.6
	pN1	100	36.9
	pN2	34	12.5
**Distant Metastases**	Absent	266	98.2
	Present	5	1.8
**AJCC/UICC TNM Stage**	I A 1	2	0.7
	I A 2	16	5.9
	I A 3	25	9.2
	I B	30	11.1
	II A	20	7.4
	II B	74	27.3
	III A	73	26.9
	III B	26	9.6
	IV A	3	1.1
	IV B	2	0.7
**Grading**	Grade 1	3	1.1
	Grade 2	139	51.3
	Grade 3	129	47.6
**Resection Status**	R0	235	86.7
	R1/R2	36	13.3
**Total**		271	100

## Supplementary Materials

The following are available online at http://www.mdpi.com/2072-6694/10/9/281/s1, Tables S1–S5: Association between the immunohistochemical markers and pathologic parameters; Figure S1: Survival curves of raw values of the immunohistochemical stainings.
