# Supplementary material for: Expression Analysis of Autophagy Related Markers LC3B, p62 and HMGB1 Indicate an Autophagy-Independent Negative Prognostic Impact of High p62 Expression in Pulmonary Squamous Cell Carcinomas
Source: Cancers (Basel). 2018 Aug 21;10(9):281. doi: 10.3390/cancers10090281 (PMC6162479; doi:10.3390/cancers10090281)

Supplementary Materials: Expression Analysis of Autophagy Related Markers LC3B, p62 and HMGB1 Indicate an Autophagy-Independent Negative Prognostic Impact of High p62 Expression in Pulmonary Squamous Cell Carcinomas

Rupert Langer, Christina Neppl, Manuel Keller, Ralph A. Schmid, Mario P. Tschan and Sabina Berezowska.

Table S1. LC3B Expression and clinico-pathologic parameters.

Parameter		LC3B		Total	*p*-Value
		Low	High		
gender	Male	104	121	225	0.627
	Female	19	27	46	
Age (median)	≤69	64	74	138	0.807
	>69	59	74	133	
pT-category	pT1a	1	3	4	0.839
	pT1b	7	12	19	
	pT1c	17	16	33	
	pT2a	23	31	54	
	pT2b	16	23	39	
	pT3	30	34	64	
	pT4	29	29	58	
pN-category	pN0	61	76	137	0.225
	pN1	42	58	100	
	pN2	20	14	34	
Distant metastases	M0	120	146	266	0.662
	M1	3	2	5	
Grading	Grade 1/2	55	87	142	0.028
	Grade 3	68	61	129	
total		123	148	271	

Table S2. p62 dot-like/cytoplasmic expression and clinico-pathologic parameters.

Parameter		p62 Dot-Like/Cytoplasmic		Total	*p*-Value
		Low	High		
gender	Male	97	128	225	0.139
	Female	14	32	46	
Age (median)	≤69	61	77	138	0.323
	>69	50	83	133	
pT-category	pT1a	1	3	4	0.236
	pT1b	10	9	19	
	pT1c	17	16	33	
	pT2a	23	31	54	
	pT2b	12	27	39	
	pT3	30	34	64	
	pT4	18	40	58	
pN-category	pN0	60	77	137	0.294
	pN1	35	65	100	
	pN2	16	18	34	
Distant metastases	M0	109	157	266	1.000
	M1	2	3	5	
Grading	Grade 1/2	65	77	142	0.108
	Grade 3	46	83	129	
total		160	271	160	

Table S3. p62 nuclear expression and clinico-pathologic parameters.

Parameter		p62 Nuclear		Total	*p*-Value
		Low	High		
gender	Male	131	94	225	0.871
	Female	26	20	46	
Age (median)	≤69	61	77	138	0.323
	>69	50	83	133	
pT-category	pT1a	3	1	4	0.576
	pT1b	12	7	19	
	pT1c	21	12	33	
	pT2a	25	29	54	
	pT2b	25	14	39	
	pT3	37	27	64	
	pT4	34	24	58	
pN-category	pN0	79	58	137	0.992
	pN1	58	42	100	
	pN2	20	14	34	
Distant metastases	M0	153	113	266	0.402
	M1	4	1	5	
Grading	Grade1/2	68	74	139	>0.001
	Grade 3	89	40	129	
total		157	114	271	

Table S4. HMGB1 expression and clinico-pathologic parameters.

Parameter		HMGB1		Total	*p*-Value
		Low	High		
gender	Male	122	103	225	0.106
	Female	31	15	46	
Age (median)	≤69	75	63	138	0.268
	>69	82	51	133	
pT-category	pT1a	0	4	4	0.126
	pT1b	13	6	19	
	pT1c	19	14	33	
	pT2a	34	20	54	
	pT2b	24	15	39	
	pT3	36	28	64	
	pT4	27	31	58	
pN-category	pN0	78	59	137	0.987
	pN1	56	44	100	
	pN2	19	15	34	
Distant metastases	M0	151	115	266	0.656
	M1	2	3	5	
Grading	Grade 1/2	85	57	142	0.270
	Grade 3	68	61	129	
total		153	118	271	

Table S5. Combination LC3B and p62 and clinico-pathologic parameters.

Parameter		LC3B/p62				Total	*p*-Value
		LL	LH	HL	HH		
gender	Male	53	51	44	77	225	0.380
	Female	6	13	8	19	46	
Age (median)	≤69	30	34	31	43	138	0.370
	>69	29	30	21	53	133	
pT-category	pT1a	0	1	1	2	4	0.447
	pT1b	3	4	7	5	19	
	pT1c	11	6	6	10	33	
	pT2a	12	11	11	20	54	
	pT2b	7	9	5	18	39	
	pT3	18	12	12	22	64	
	pT4	8	21	10	19	58	
pN-category	pN0	33	28	27	49	137	0.344
	pN1	15	27	20	38	100	
	pN2	11	9	5	9	34	
Distant metastases	M0	58	62	51	95	266	0.818
	M1	1	2	1	1	5	
Grading	Grade 1/2	31	24	34	53	142	0.023
	Grade 3	28	40	18	43	129	
total		59	64	52	96	271	

Figure S1. Survival curves of raw values of the immunohistochemical stainings.

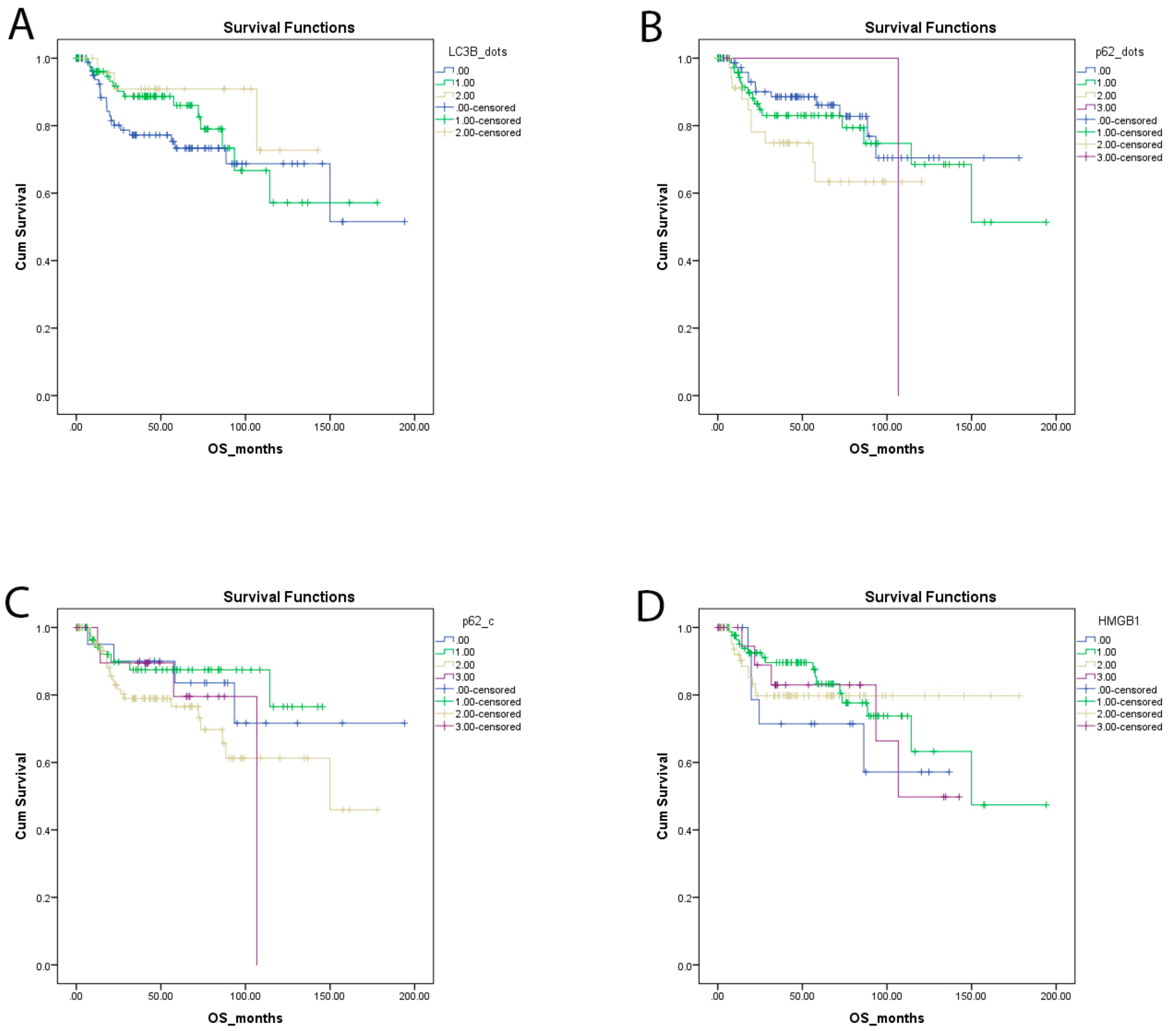

Supplement: Supplementary file 1 [file cancers-10-00281-s001.pdf]
